# Global biochemical profiling of fast-growing Antarctic bacteria isolated from meltwater ponds by high-throughput FTIR spectroscopy

**DOI:** 10.1371/journal.pone.0303298

**Published:** 2024-06-17

**Authors:** Volha Akulava, Valeria Tafintseva, Uladzislau Blazhko, Achim Kohler, Uladzislau Miamin, Leonid Valentovich, Volha Shapaval

**Affiliations:** 1 Faculty of Science and Technology, Norwegian University of Life Sciences, Ås, Norway; 2 Faculty of Biology, Belarusian State University, Minsk, Belarus; 3 Institute of Microbiology, National Academy of Sciences of Belarus, Minsk, Belarus; Universidade Estadual de Ponta Grossa, BRAZIL

## Abstract

Fourier transform infrared (FTIR) spectroscopy is a biophysical technique used for non-destructive biochemical profiling of biological samples. It can provide comprehensive information about the total cellular biochemical profile of microbial cells. In this study, FTIR spectroscopy was used to perform biochemical characterization of twenty-nine bacterial strains isolated from the Antarctic meltwater ponds. The bacteria were grown on two forms of brain heart infusion (BHI) medium: agar at six different temperatures (4, 10, 18, 25, 30, and 37°C) and on broth at 18°C. Multivariate data analysis approaches such as principal component analysis (PCA) and correlation analysis were used to study the difference in biochemical profiles induced by the cultivation conditions. The observed results indicated a strong correlation between FTIR spectra and the phylogenetic relationships among the studied bacteria. The most accurate taxonomy-aligned clustering was achieved with bacteria cultivated on agar. Cultivation on two forms of BHI medium provided biochemically different bacterial biomass. The impact of temperature on the total cellular biochemical profile of the studied bacteria was species-specific, however, similarly for all bacteria, lipid spectral region was the least affected while polysaccharide region was the most affected by different temperatures. The biggest temperature-triggered changes of the cell chemistry were detected for bacteria with a wide temperature tolerance such *Pseudomonas lundensis* strains and *Acinetobacter lwoffii* BIM B-1558.

## Introduction

During the last decade, Fourier transform infrared (FTIR) spectroscopy became a standard analytical technique for comprehensive biochemical profiling of microorganisms [1–6]. FTIR spectroscopy allows identifying main biomolecules present in microbial biomass, including proteins, lipids, carbohydrates, and nucleic acids [4,7]. Each biomolecule has specific functional groups that possess vibrational modes with unique spectral signatures when assessed by FTIR [8]. Therefore, FTIR spectroscopy has been suggested as a powerful tool for compositional and structural analysis of microbial biomass. For example, by examining protein spectral region 1700–1500 cm^–1^ details on protein’s secondary structure such as presence of α-helices or β-sheets can be obtained [9]. It has been shown that FTIR spectra can be used for the estimation of relative total lipid content and its changes in oleaginous microorganisms [10–12]. Further, numerous studies reported successful application of FTIR spectroscopy for the identification of microorganisms, where it has been shown that FTIR biochemical signatures of different bacteria often reflect their phylogenetic relationships [13,14]. FTIR spectroscopy can contribute to understanding molecular underpinnings of phenomena like adaptive tolerance responses of bacteria when they are subjected to various environmental stress conditions [15,16]. FTIR spectroscopy for the characterization and identification of bacteria has been employed since the 90s. [17–24]. Numerous studies have been done on the characterization of bacterial metabolites such as lipidic compounds [25,26], exopolysaccharides [27], biosurfactants [28], enzymes [29] as well as bacterial processes such as fermentation [30,31], bioremediation [32], degradation of feathers [29], biodegradation of colored wastewater [33], degradation of plastics [34] and petroleum materials [35].

The FTIR technique has a minor destructive effect on cells and allows their total biochemical profiling in nearly intact form. The typical protocol to prepare microbial cells for high-throughput screening (HTS) FTIR includes: (i) cultivation step to obtain enough amount of microbial biomass, (ii) washing of microbial cells to remove medium components which may interfere with biomass signals on the FTIR spectra, (iii) depositing a small amount of cell suspension (8–10 μl) on the FTIR silica plate with subsequent drying at room temperature before measurements [17]. This preparation protocol can be readily automated [36,37] and the throughput can be increased by using microtiter plates [38–41]. Thus, FTIR analysis can be done in a high-throughput setting, which is advantageous for biochemical phenotyping of newly isolated microorganisms and biotechnological screenings [42–45]. Therefore, FTIR spectroscopy has been positioned as a Next-Generation Phenotyping (NGP) technique for building chemotaxonomic maps of existing microbes, identification and characterization of newly isolated [45,46].

Recently, we successfully applied FTIR spectroscopy for biochemical characterization and bioprospecting of green snow Antarctic bacteria [29,47,48]. In one of the studies, we have shown that green-snow Antarctic bacteria cultivated in two forms of culture medium–semi-solid agar and broth and at different temperatures possessed considerable differences in cell chemistry [47]. These biochemical cellular differences were associated with the changes across all spectral regions of the FTIR spectrum: (i) lipid region 3050–2800 cm^-1^ and 1700–1800 cm^-1^ indicating changes in membrane lipids and some storage ester-based compounds such as polyhydroxyalkanoates (PHAs), (ii) protein region 1700–1500 cm^-1^ providing information on the protein structure, (iii) mixed region 1500–1200 cm^-1^ where the information about proteins, lipids and phosphorus compounds is reflected, (iv) polysaccharide region 1200–700 cm^-1^ reflecting information about cell wall and storage polysaccharides and (v) so-called fingerprint region at 900–700 cm^-1^ consisting of mainly peaks without any special assignment but very characteristic for different microbial strains [4]. In this study, the analysis of the mentioned above spectral regions were employed for biochemical characterization and bioprospecting of meltwater pond bacteria.

Overall main aim of the present study was to perform global biochemical characterization of newly isolated bacteria from Antarctic meltwater temporary ponds and evaluate cellular biochemical changes in bacterial cells when grown in different culture forms and temperatures by high-throughput FTIR spectroscopy.

## Materials and methods

### Bacterial strains

Twenty-nine fast-growing Antarctic bacteria from the Belarussian Collection of Non-pathogenic Microorganisms (Institute of Microbiology of the National Academy of Science of Belarus) were used in the study. The bacteria are Gram-positive and Gram-negative, psychrotrophic, and belong to seventeen species. The bacteria were isolated from water samples collected during the 5th Belarusian Antarctic Expedition in the austral summer season (January 2013) from the middle part of the water column of nine non-flowing temporary meltwater ponds (TMPs) located in rock baths of the Vecherny region of the Thala Hills oasis in the central part of Enderby Land (East Antarctica). Identification by 16S rRNA gene sequencing and comprehensive physiological characterization (enzymatic activity, optimal growth temperature, and antibiotic resistance, pigments characterization) of the isolates were previously reported [49–51].

### Experiment design and cultivation conditions

For the biochemical profiling by FTIR spectroscopy, bacteria were cultivated on brain heart infusion agar (BHIA) and broth (BHIB) (Sigma Aldrich, USA) at 18°C. Rich complex media were chosen due to the limited growth of bacteria on minimal media. The cultivation temperature of 18°C was selected based on the screening experiments previously performed [47–49]. Cultivation on BHIA was performed for 3–5 days, depending on the isolate, to obtain enough biomass for FTIR measurements. Cultivation in BHIB was performed at 18°C for 3 days for all isolates in the Duetz Microtiter Plate System–Duetz-MTPS (Enzyscreen, Heemstede, Netherlands), consisting of 24-square low polypropylene deep-well plates, low-evaporation sandwich covers, and extra high cover clamp system as was previously described [38,42–44,52,53]. Cultivation media and Duetz-MTPS were sterilized by autoclaving at 121°C for 15 min before inoculation. The autoclaved MTPS were filled with 3 mL of sterile broth medium per well, and each well was inoculated with a single colony of fresh cultures prepared on BHIA. For the sterility control, one well in each microtiter plate was filled with the medium without inoculation. Duetz-MTPS were mounted on the shaking platform of MAXQ 4000 shaking incubator (Thermo Fisher Scientific, Waltham, MA, USA) and incubated for 3 days at 18°C with 370 rpm agitation speed (1.9 cm circular orbit). For each bacterial isolate and media, cultivations were done in three biological replicates which were prepared from separate Petri dishes and MTPS and performed as independent experiments.

To evaluate the effect of temperature on the total cellular biochemical profile, bacterial isolates were cultivated at 4, 10, 18, 25, 30, and 37°C on BHIA. The cultivation time was for 1–12 days depending on the cultivation temperature and strain growth ability (S1 Table). The cultivations were performed in two independent biological replicates for each bacterial isolate and temperature.

### Preparation of bacterial biomass for FTIR measurements

Bacterial biomass was separated from the supernatant by centrifugation (Heraeus Multifuge X1R, Thermo Scientific, Waltham, MA, USA) at 25.200 g at 4°C for 30 min and washed with distilled water three times. Further, at the last washing step, 100–500 μL of distilled water was added to the cell pellet and re-suspended. 10 μL of the homogenized bacterial suspension were pipetted onto the IR-light-transparent silicon 384-well silica microplates (Bruker Optics GmbH, Ettlingen, Germany). Supernatant samples were diluted ten times with distilled water and were pipetted onto the IR-light-transparent silicon 384-well silica microplates. Both, samples of bacterial suspension and supernatant were pipetted in three technical replicates and dried at room temperature for at least 1 hours before the analysis.

### FTIR spectroscopy analysis

FTIR transmittance spectra were measured using a high-throughput screening extension unit (HTS-XT) coupled to the Vertex 70 FTIR spectrometer (both Bruker Optik, Germany). The FTIR system was equipped with a globar mid-IR source and a deuterated L-alanine doped triglycine sulfate (DLaTGS) detector. The HTS-FTIR spectra were recorded with a total of 64 scans, using Blackman-Harris 3-Term apodization, spectral resolution of 6 cm^-1^, and digital spacing of 1.928 cm^-1^, over the range of 4000–400 cm^-1^, and an aperture of 6 mm. The ratio of a sample spectrum to a spectrum of the empty IR transparent microplate was used to calculate a final spectrum. Background spectra of the silica microplate were collected prior to each sample measurement to account for variations in water vapor and CO_2_. Generated transmittance spectra were exported for further analysis. Each sample was analyzed in three technical replicates. For data acquisition and instrument control, the OPUS software (Bruker Optik GmbH, Germany) was used.

### Estimation of chemical variability

Chemical variability of bacterial biomass produced at different conditions was estimated using FTIR data. The samples were grouped by several criteria: (1) technical and biological replicates, (2) cultivation conditions such as time, temperatures, and media and (3) taxonomic units such as strain, specie and genus. In addition, chemical variability was calculated for different spectral regions: lipid region at 3050–2800 cm^-1^ combined with ester region 1800–1700 cm^-1^, protein region at 1700–1500 cm^-1^, mixed region at 1500–1200 cm^-1^, and polysaccharide region at 1200–700 cm^-1^. Variability was calculated for all data together and separately for data acquired from agar and broth cultivations. Chemical variability of spectra within a group was estimated by median distance from a sample to the center of the group. The center of the group was calculated as a mean of all spectra within the group. The distance between spectra was calculated as 1 Pearson’s correlation coefficient (PCC). The closer this value is to 0, the more similar the individual spectrum is to the mean spectrum, indicating lower variability. As some categories may include several groups (e.g. 17 species), the variability was calculated first for each group and then averaged. The raw spectra were utilized without modification as they showed no visible scattering distortions, only variations in the constant baseline and multiplicative effects. These changes do not impact the Pearson correlation analysis.

### Spectral preprocessing and multivariate data analysis

Prior to data analysis, the spectra were preselected using a quality test developed by Tafintseva et al. [45,54,55]. The spectra that passed the quality test were preprocessed in the following way: (1) averaging of technical replicates for each sample; (2) second derivative using Savitzky−Golay algorithm with the second order polynomial and different window sizes that were selected depending on the spectral region– 11 points for lipids region, 21 points for protein region, 15 points for mixed region and 13 for carbohydrate region, 11 points when the whole spectral region was used; (3) selecting spectral regions of interest: 3100–2800 cm^-1^ and 1800–1700 cm^-1^ for lipids, 1700–1500 cm^-1^ for proteins, 1500–1200 cm^-1^ as mixed region and 1200–700 cm^-1^ for polysaccharides or using the whole spectral region 3100–700 cm^-1^ (4) extended multiplicative signal correction (EMSC) with linear and quadratic terms in order to separate informative signals from spectral artefacts and minimize variability due to light scattering or sample thickness [39,55–59]. All datasets were preprocessed as listed above.

After preprocessing, multivariate data analysis techniques, such as principal component analysis (PCA), were applied. This analysis aimed to examine the total cellular biochemical profile of bacteria. Its goals were to unveil underlying patterns, visually represent data points in fewer dimensions while retaining maximum information and investigate relationships among dependent variables [60].

Further, using correlation loading plots the effect of temperature was investigated. For the correlation loading plots, PCA model built on the whole spectral region was used and a set of preselected peaks listed in Table 2 were visualized. Since variability between different genera was higher than variability between temperatures, correlation analysis was done for each species separately.

The Unscrambler, V10.01 (CAMO PROCESS AS, Oslo, Norway) and algorithms in Matlab, V23.a (The Mathworks, Inc., Natick, MA) as well as Orange data mining toolbox version 3.31.1 (University of Ljubljana, Ljubljana, Slovenia) were used to perform the all analysis [61,62].

## Results

### Variability of the total cellular biochemical profile

Given the experiment’s design, which included several variables such as cultivation time, temperature, media, and culture, all known to influence the total cellular chemical composition of the biomass, assessing the introduced variability by these dimensions is essential. The variability was estimated using Pearson’s distance to the middle of the group’s cluster, and the results are presented in Table 1. It can be seen that agar cultivations resulted in less total cellular chemical variability of bacterial biomass than liquid culture cultivations (Table 1). The highest chemical variability was observed between different genera followed by species and strains, while the lowest variability was for biological and technical replicates. The variability between strains of the same specie was much higher than the variability in biological and technical replicates (Table 1). The polysaccharide spectral region exhibited the greatest variability across all tested taxonomic levels, while the lowest variability was observed for lipid region for biomass obtained from agar and proteins for biomass obtain from broth cultivations. Temperature showed different impact on the total cellular chemical composition at different spectral regions, for example, an increase in temperature resulted in a higher variability for carbohydrate, lipid and mixed regions, while protein region showed increase in variability when extreme temperatures such us 4 and 37°C were used for the cultivations (Table 1). Variability between cultivation days was lower than variability between cultivation temperatures.

**Table 1:** Variability and reproducibility of the total cellular biochemical profile analysed by FTIR spectroscopy.

**Variability estimated as (1-PCC)10^3**					
**Variability**	**Spectral region**				
	**whole**	**lipids**	**proteins**	**mixed**	**carbs**
	**4000–700 cm** ^**-1**^	**3050–2800 cm** ^-1^ **1800–1700 cm** ^-1^	**1700–1500 cm** ^**-1**^	**1500–1200 cm** ^**-1**^	**1200–700 cm** ^**-1**^
**Cultivation in BHIB at 18°C**					
Technical replicates	0,4	0,1	0,4	0,2	0,5
Biological replicates	8,2	6,2	5,6	5,2	24,8
Strain	17,1	8,0	7,9	9,7	48,9
Species	16,6	11,4	7,1	11,6	47,5
Genus	40,0	23,6	17,2	25,1	111,7
**Cultivation in BHIA at 18°C**					
Technical replicates	0,7	0,2	0,6	0,5	1,0
Biological replicates	3,6	1,6	2,7	2,9	5,1
Strain	7,4	2,9	2,2	6,4	25,3
Species	10,3	5,0	4,9	12,0	40,8
Genus	32,8	27,5	10,3	26,6	58,6
**Cultivation at different temperatures on BHIA**					
Technical replicates	0,7	0,4	0,5	0,5	1,2
Biological replicates	3,1	1,6	2,0	2,3	5,3
Cultivation time (days)	7,9	4,2	3,8	6,7	17,3
Strain	10,0	4,9	4,0	8,9	32,5
Species	17,8	7,9	5,6	17,2	56,6
Genus	34,2	28,5	11,3	27,8	75,3
4°C	33,0	32,7	8,7	27,9	46,1
10°C	41,2	32,3	8,3	25,9	57,8
18°C	32,8	27,5	10,3	26,6	58,6
25°C	32,1	23,1	12,6	24,2	88,0
30°C	22,2	16,1	12,4	16,7	91,2
37°C	10,6	6,6	8,4	7,7	84,1

### Biochemical profile of Antarctic meltwater bacteria grown on agar and broth

**Fig 1:**
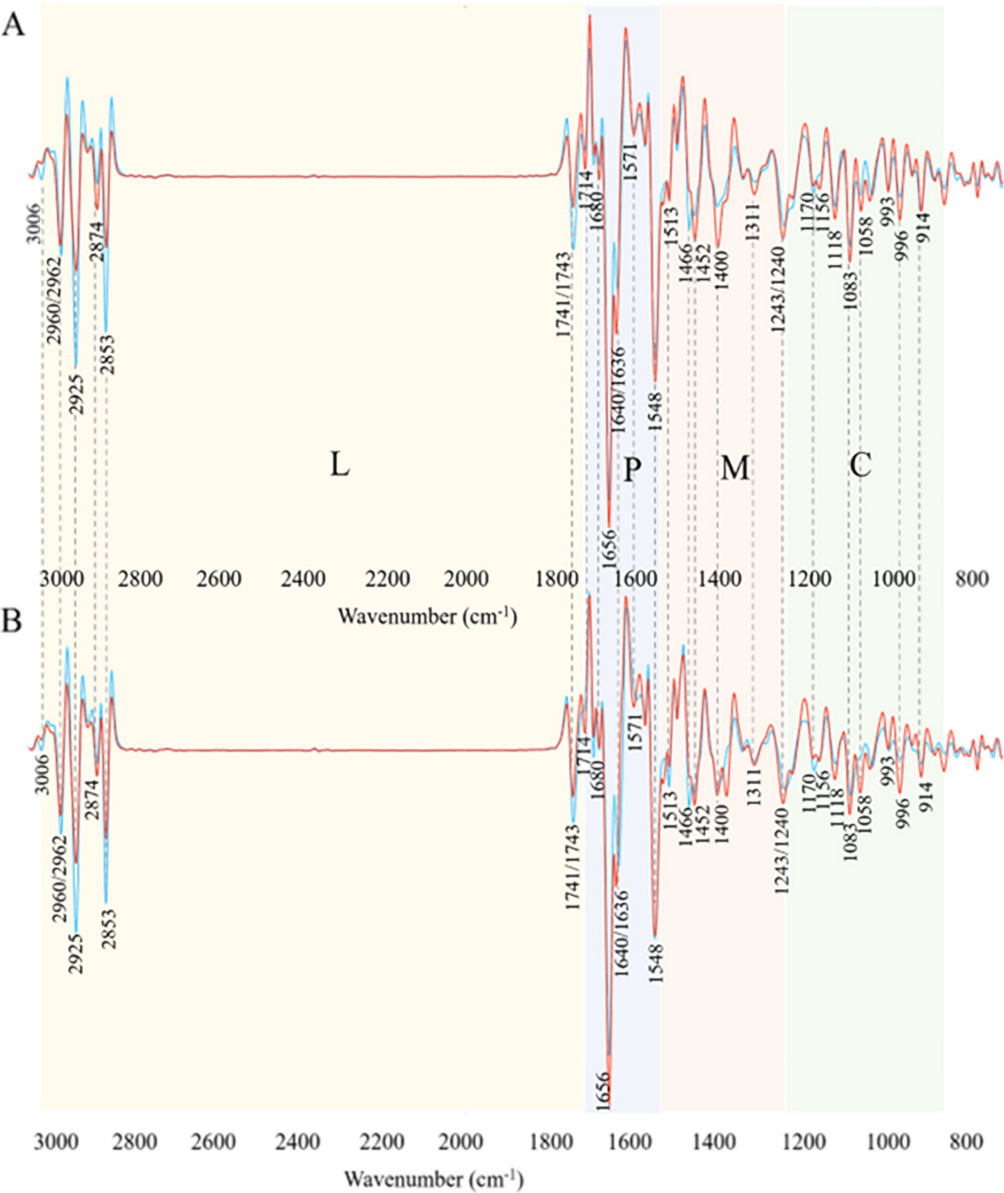
Second derivative spectra of Gram-positive (red) and Gram-negative (blue) Antarctic bacteria grown on (A) BHIA, (B) BHIB. Colors and letters represent regions: L-lipid/ester region, P-protein region, M-mixed region, C-carbohydrate region. Peak assignment given in Table 2.

Total cellular biochemical profile of the studied Antarctic meltwater bacteria using FTIR spectroscopy was first evaluated when bacteria were grown on agar and in liquid BHI media at 18°C. Fig 1 shows averaged second derivative spectra of Gram-positive and Gram-negative bacteria grown on agar and broth. The BHI broth, a rich and complex medium, might contain lipidic compounds affecting bacterial lipid profiles. However, FTIR spectroscopy analysis showed no lipid-related peaks as was shown in our previous findings [51]. The assignment of the main characteristic peaks and their misalignment for Gram-positive and Gram-negative bacteria are given in Table 2.

**Table 2:** Peaks assignment for the FTIR-HTS spectra of Antarctic bacteria. Peak frequencies have been obtained from the second derivative spectra. Abbreviations: Asym, antisymmetric; sym, symmetric; str, stretching; def, deformation [17–19,25,63–69].

Wavenumber (cm^-1^)		Molecular vibration	Cell component	Ref.
Gram -	Gram+			
**Lipid region 3050**–**2800 cm**^**-1**^ **+ 1800**–**1700 cm**^**-1**^				
3006		= C-H stretching	Polyunsaturated lipids	[18,67]
2960	2962	-C-H (CH_3_) stretching	Mainly unsaturated lipids, little contribution from proteins, carbohydrates, nucleic acids	[17,18,25,66,69]
2925		-C-H (CH_2_) stretching		
	
2875		-C-H (CH_3_) stretching		
2853		-C-H (CH_2_) stretching		
1742		>C = O stretching	Acyl glycerides, esters, lipids	[17,19,25,65,66]
1714		νC = O stretching	Esters, carboxylic acids	[65,66,69]
**Protein region 1700**–**1500 cm**^**-1**^				
1693, 1680		-C = O stretching	Antiparallel pleated sheets of amide I band	[65,66,69]
1656		-C = O stretching	amide I of a-helical structures	[17,18,25,65,66,69]
1636	1640	-C = O stretching	amide I of β-pleated sheet structures	[18,19,25,65,66,69]
1570		νCOO asym	Asparatate, glutamate	
1548		CONH bending	Amid II	[65,66]
1513		Benzene ring stretch	Aromatic amino acids (Phe, Tyr, Trp)	[19,66]
**Mixed region/ 1500**–**1200 cm**^**-1**^				
1466	-	CH_2_ deformation	mainly lipids with little contributions from protein (membrane lipids), phospholipids	[17,19,25,65,66]
-	1453	CH_3_ deformation		
1400		C = O symmetric stretching of COO^-^	Amino acids, fatty acyl chains	[66,69]
1311		C-N	amide III band	[66]
1240	1243	P = O asymmetric stretching of >PO_2_	Phosphodiesters, phospholipids (membrane), teichoic acids, lipoteichoic acids (cell wall), nucleic acids (nucleoid) mainly nucleic acids with the little contribution from phospholipids	[19,65]
1222	1220			
**Carbohydrate region 1200**–**700 cm**^**-1**^				
1170		C-O, C-C str., C-O-H, C-O-C def.	Carbohydrates, glycogen and nucleic acids	[17,19,65,69]
1155				
1119				
1082		C-O stretching of glycogen PO_2_^˗^ symmetric stretching	Phosphodiesters, phospholipids (membrane), nucleic acids (nucleoid), teichoic acids (peptidoglycan), glycogen	[17–19,25,65,66,69]
1059	1058	PO_2_ str. and C-O-H str.	Phosphate ester. and oligosaccharides	[69]
1037	1030	C-O, C-C str., C-O-H, C-O-C def.	Carbohydrates	[19,65]
1045–1025		O stretching	O stretching of glycogen	[63]
964		C-O, C-C str., C-O-H, C-O-C def.	Carbohydrates, nucleic acids	[19,65]
993		νC-O ribose, νC-C	Ribose skelet (ARN) ribosomes, sugars	[69]
**‘‘fingerprint region” 900**–**400 cm**^**-1**^				

A visual comparison of FTIR biochemical spectral profiles revealed distinct chemical differences which are related to taxonomy of the studied bacteria and/or growth medium. Several shifts for characteristic peaks were observed on the spectra of bacteria from different Gram groups. A slight peak shift was detected for -CH_3_ group which was from 2960 cm^-1^ for Gram-negative bacteria to 2962 cm^-1^ for Gram-positive bacteria (Fig 1A and B, Table 2). Also, another peak shift was detected for the ester peak, where it was at 1743 cm^-1^ for Gram-positive bacteria and at 1741 cm^-1^ for Gram-negative (Fig 1 and Table 2). All Gram-negative bacteria grown on agar and in broth media showed higher absorbance values for all lipid peaks compared to Gram-positive bacteria, indicating of a higher total lipid content in their cells (Fig 1A and 1B). The averaged spectrum of Gram-negative bacteria had elevated lipid peaks at 3006 cm^–1^, 2925 cm^–1^, 2853 cm^–1^ and 1741 cm^–1^ indicating a higher content of unsaturated, saturated lipids and polyesters, respectively (Fig 1A and 1B). Further, a peak at 1466 cm^-1^ related to C-H deformation/scissoring of -CH_2_ group mainly in lipids with a little contribution from proteins was detected on the averaged spectrum of Gram-negative bacteria and it was absent for the Gram-positive bacteria cultivated on agar and broth (Fig 1A and 1B). While averaged spectrum of Gram-positive bacteria showed a higher absorbance for the peak at 1452 cm^-1^ related to -CH_3_ deformation in lipids. (Fig 1A and 1B, Table 2).

Proteins are the major biochemical components of bacterial cells, therefore, typically they are represented by the peaks with the highest absorbance in the region 1700–1500 cm^-1^. This was also observed for the studied Antarctic bacteria grown on agar and broth media, where the most characteristic protein peaks were C = O stretching vibrations in amino acids (amide I) at 1656 cm^-1^ associated with α-helical structures, peak at 1636/1640 cm^-1^ associated with β-pleated sheet structures, peak at 1548 cm^-1^ related to N-H deformation vibrations (amide II) and the peak at 1311 cm^-1^ associated with C-N vibrations of amide III bond. The main differences in the protein region for the bacteria grown on agar and in broth were related to the lower absorbance of protein peaks in Gram-negative bacteria and appearance of a shift for the C = O stretching amide I peak at 1636 cm^-1^ for Gram-negative to 1640 cm^-1^ for Gram-positive (Fig 1A and 1B, Table 2).

Further, some differences between Gram-positive and Gram-negative bacteria grown on agar and in broth were observed in mixed spectral region 1500–1200 cm^-1^ and polysaccharide spectral region 1200–700 cm^-1^. Peaks at 1400 cm^-1^, 1240 cm^-1^ associated with phosphodiester group present in various molecules, such as DNA, phospholipids and teichoic acids and lipoteichoic acid and had higher absorbance values for Gram-positive bacteria, peak at 1170 cm^-1^ had higher absorbance values for the spectra of Gram-negative bacteria. For the spectra of Gram-positive bacteria, the peak at 1156 cm^-1^ associated with C-O, C-C stretching., C-O-H, C-O-C deformation in carbohydrates had higher absorbance compared to Gram-negative bacteria (Fig 1A and 1B, Table 2). Interestingly, difference between Gram-groups in the carbohydrate region notably increases when bacteria were grown in broth medium compared to agar medium.

**Fig 2:**
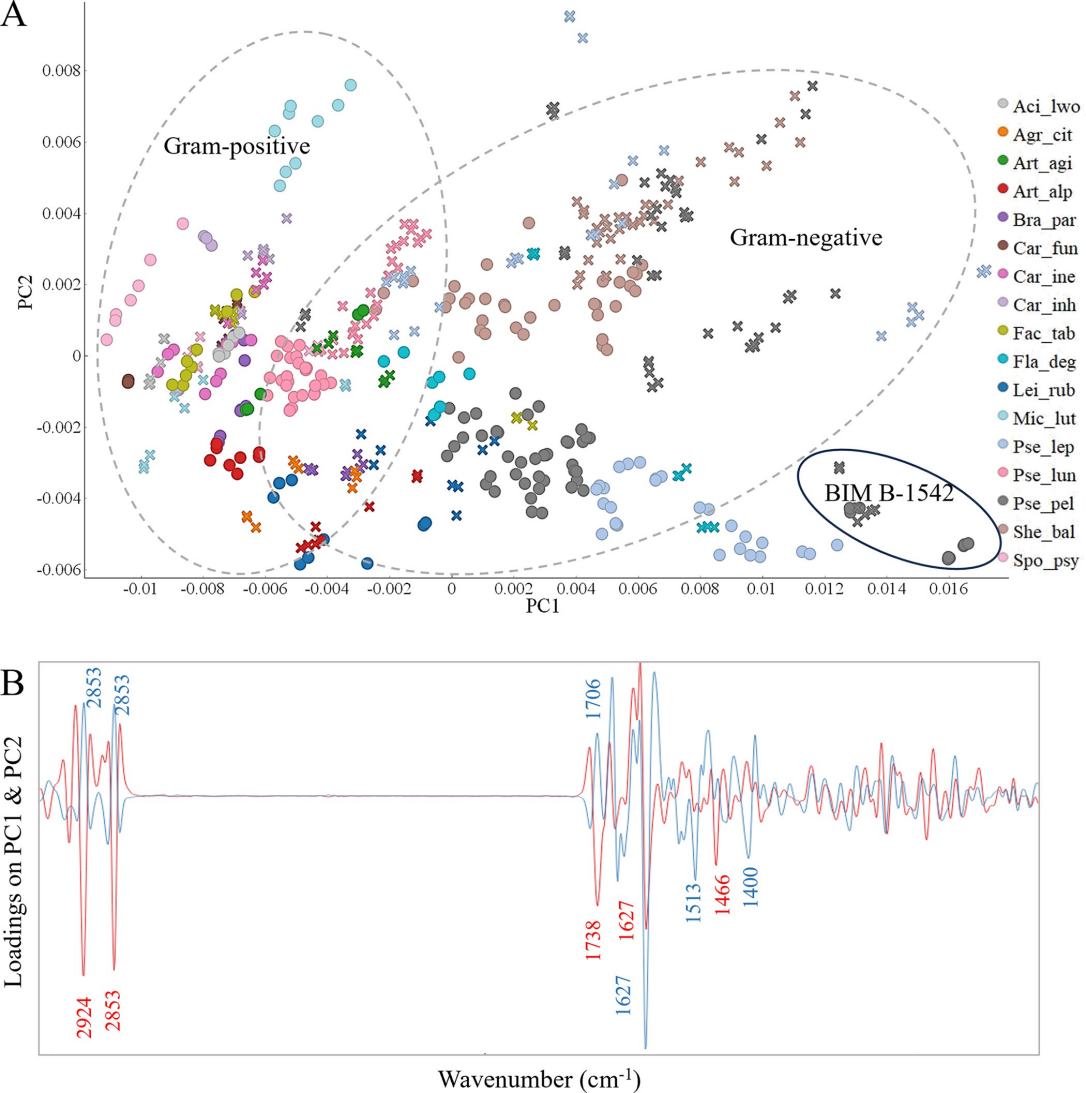
Principal component analysis (PCA) of the preprocessed FTIR spectra of Antarctic bacteria grown in different media (‘●’–Agar, ‘Ñ’–Broth) at 18°C. A–Score plot of PC1 and PC2 components, colors represent genera, shapes represent cultivation temperatures. B–Loading plot of FTIR data with main contributing peaks, PC1 (red) and PC2 (blue). PC1 provided 53% of explained variance and PC2 provided 12% of explained variance.

Following the visual comparison reported above, the preprocessed FTIR spectra of bacterial biomass underwent PCA analysis to explore the connections between the biochemical profiles of the studied bacteria cultivated on various forms of BHI medium. PCA score and loading plots for the whole spectral region are displayed in Fig 2A and 2B, respectively. It can be seen that samples of Gram-negative bacteria except *Acinetobacter lwoffii* BIM B-1558 and *Pseudomonas lundensis* isolates are located in the area of positive PC1 score indicating a higher lipid content in the cells of these bacteria, while most of the samples of Gram-positive bacteria are located in the area of negative PC1 scores, meaning higher protein content in the cells (Fig 2A). The most significant peaks, identified on the loading plot, to be responsible for the distribution of samples along the PC1 axis are lipid peaks associated with (i) chain length (-CH_2_ stretching at 2924 cm^-1^ and 2853 cm^-1^ and -CH_2_ bending at 1466 cm^-1^), (ii) relative total content of lipidic compounds (C = O stretching at 1738 cm^-1^) and protein peaks associated with proteins’ structure (-C = O stretching at 1627 cm^-1^) (Fig 2B) or the PC2 axis, the following peaks were registered on the loading plot as significant: peaks associated with the -C = O stretching in proteins at 1627 cm^-1^, 1513 cm^-1^ and 1400 cm^-1^. Thus, a separation along the PC2 axis is mainly due to the proteins. Both PC1 and PC2 appear to be responsible for the dissimilarities between different bacterial species cultivated on different media forms: agar and broth (Fig 2A).

Analysis of clustering at the genus level was done only for three genera *Pseudomonas*, *Arthrobacter*, and *Carnobacterium*. The remaining genera were represented by only a single species, and, therefore, no conclusion could be drawn. Genera represented by two or more species are discussed below. The total cellular biochemical profile of bacteria from genera *Carnobacterium* showed to be little affected by the cultivation media. Interestingly, bacteria from genus *Carnobacterium* did not show a big variation in biochemical profile for different species cultivated on different media. It can be seen from the PCA score plot that species-specific variability inside of genera *Pseudomonas* and *Arthrobacter* is high, and each specie forms a separate cluster for cultivation on agar and in broth media. *Pseudomonas peli* BIM B-1542 grown on agar and in broth clustered outside of other *Pseudomonas* strains and can be characterized by a higher lipid content (Fig 2A).

To uncover species-specific differences in biochemical profile of the studied bacteria, visual comparison of the preprocessed FTIR spectra was performed for each of the species separately and the results are presented on the S1 Fig. It could be seen that the most pronounced effect of cultivation media on the total cellular biochemical profile was detected for Gram-positive bacteria from phylum Actinobacteria, especially it was visible for *Micrococcus luteus* BIM B-1545. Further, it can be seen that the lipid region is little affected by the cultivation media and visible changes were observed only for *Pseudomonas peli* strains for the peaks related to -CH_2_ stretching in lipids at 2935 cm^-1^ and 2853 cm^-1^ and esters at 1741 cm^-1^ and for *Micrococcus luteus* BIM B-1545 similar effect was observed for the peaks related to -CH_3_ stretching in lipids at 2960 cm^-1^ and 2875 cm^-1^ and esters at 1743 cm^-1^. In the protein region, changes in intensity of amide I band at 1565 cm^-1^, 1636 cm^-1^ and amide II at 1548 cm^-1^ were slightly higher for the bacteria cultivated on agar for Gram-positive bacteria and lower for Gram-negative. Additionally, slight shift to lower wavenumbers was detected for amide I peak at 1640 cm^-1^ related to β-sheet structures of proteins on broth media compared to agar media for Gram-negative bacteria. In the mixed region the highest effect was observed for *Micrococcus luteus* BIM B-1545 and bacteria related to *Arthrobacter* genus for the peaks related to -CH_2_ bending in lipids with little contributions from proteins (membrane lipids) at 1400 cm^-1^ and in vibrational modes of the phosphate groups at 1240 cm^-1^.The polysaccharide region was shown to be the most affected by cultivation media and numerous changes in polysaccharides were recorded for *Micrococcu*s *luteus* BIM B-1545, *Leifsonia* sp. BIM B-1567 and *Arthrobacter agilis* BIM B-1543 (S1 Fig).

**Fig 3:**
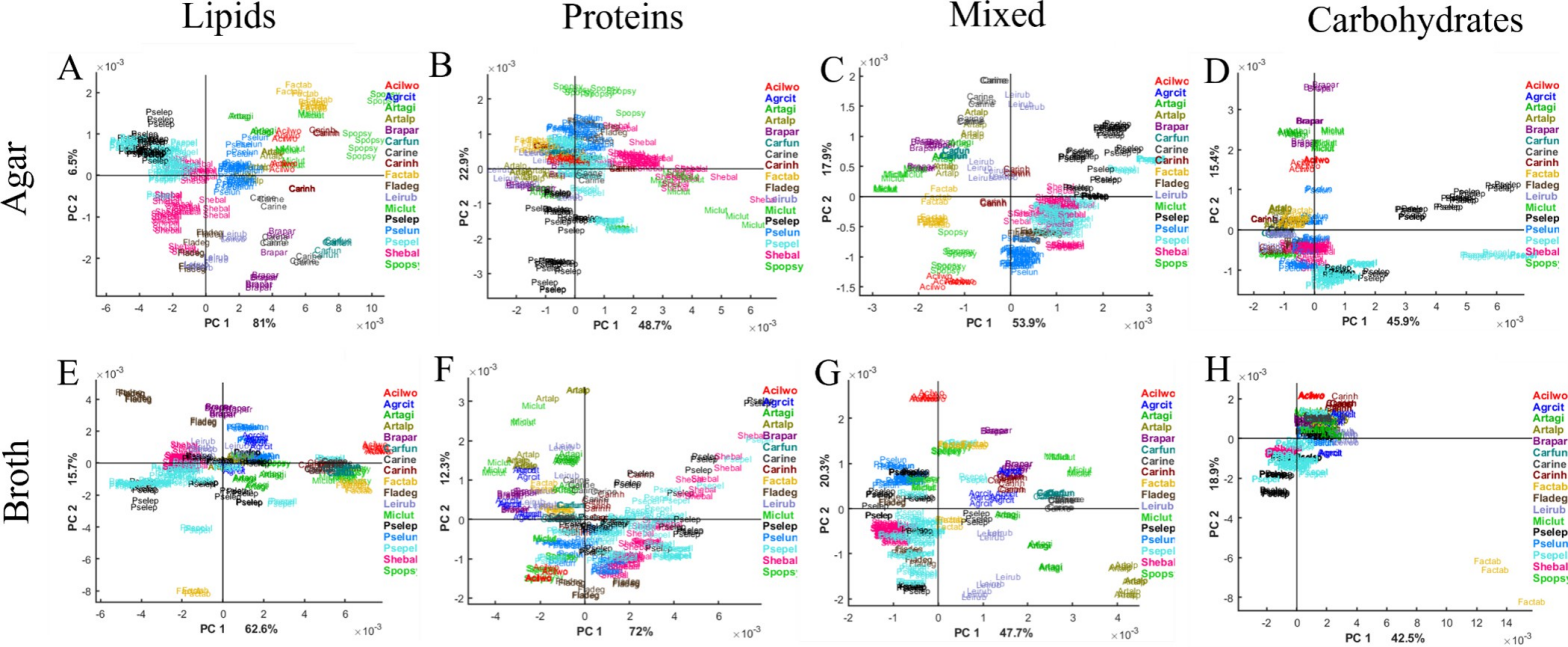
PCA score plots of normalized spectra of lipid (A, E), protein (B, F), mixed (C, G) and polysaccharide (D, H) spectral regions of the Antarctic meltwater bacteria cultivated on BHIA (A-D) and BHIB (E-H). Different colors correspond to different genera and short abbreviations given in S1 Table.

**Fig 4:**
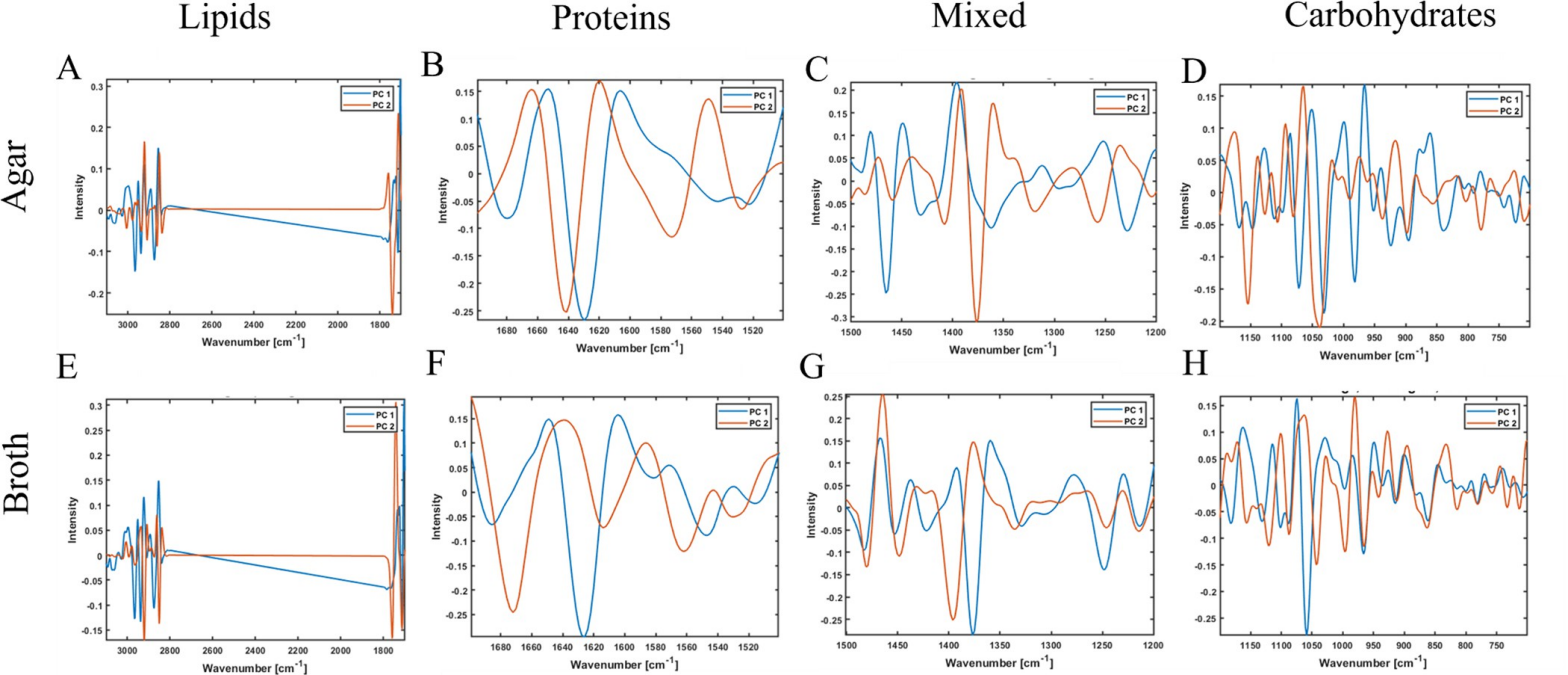
PCA loading plots of PC1 and PC2 components of normalized spectra of lipid (A, E), protein (B, F), mixed (C, G) and polysaccharide (D, H) spectral regions of Antarctic meltwater bacteria cultivated on BHIA (A-D) and BHIB (E-H).

To study the effect of the cultivation media on the total cellular biochemical profile of the bacteria, PCA analysis was done using lipid, protein, mixed, and carbohydrate regions. The changes introduced by the growth conditions were the most pronounced in the lipid region. Better clustering according to the taxonomy was observed for agar-cultivated bacteria than for broth (Fig 3A and 3E). For example, bacteria from genera *Carnobacterium* and *Micrococcus* separated well from each other after cultivation on agar, while showed more overlapping on broth medium. Some bacteria showed more discriminative clustering after being grown in broth than on agar, for example, *Flavobacterium degerlachei* BIM B-1562, *Acinetobacter lwoffii* BIM B-1558, *Brachybacterium paraconglomeratum* BIM B-1571 showed FTIR profiles overlapping with other strains when grown on agar and formed separate clusters after cultivation in broth (Fig 3E). The clustering on the PCA score plot of the lipid region is defined by the same lipid peaks as in PCA of the whole spectral region with addition of peak at 1714 cm^-1^ indicating the presence of free fatty acids (Fig 4A and 4E).

The PCA analysis of the protein region showed a high level of similarity between many bacteria from different genera and species, with clear clusters of Gram groups (Fig 3B). The following bacteria cultivated on agar exhibited relatively distinct clustering when protein spectral region was used: (i) *Sporosarcina* sp BIM B-1539, *Micrococcus luteus* BIM B-1545, (ii) all strains related to *Pseudomonas leptonychotis* and *Shewanella baltica* (Fig 3B). Cultivation in broth resulted in a relatively high variation of protein profile between different bacteria and even biological replicates (Fig 3F). The observed distribution of strains on the PCA score plot when using protein spectral region was based on the contribution from peaks at 1636 cm^-1^ and 1656 cm^-1^ related to β-pleated sheet and a-helical structures, respectively, and -C = O stretching amide I peak at 1680 cm^-1^ related to antiparallel pleated sheets (Fig 4B and 4F).

The PCA analysis of the mixed spectral region showed a clear clustering according to Gram groups, genus and species taxonomy for bacteria grown on agar and in broth. The loading plot of PC1 indicates that clustering according to Gram groups is defined by the lipid-related peaks associated with -CH_2_ stretching at 1463 cm^-1^ and C = O symmetric stretching in amino acids and fatty acyl chains (peptidoglycan) at 1400 cm^-1^ (Fig 4C and 4G).

The PCA analysis of the polysaccharide spectral region showed distinctive clustering of several agar-cultivated bacterial species, for example, *Pseudomonas leptonychotis* strains and *Acinetobacter lwoffii* BIM B-1558, *Arthrobacter agilis* BIM B-1543, *Brachybacterium paraconglomeratum* BIM B-1571 and *Micrococcus luteus* BIM B-1545 (Fig 3D and 3H). The peaks at 1082 cm^-1^, 1060 cm^-1^, 1037 cm^-1^, 970 cm^-1^ responsible for this separation are the ones related to C-O, C-C, C-O-C, P-O-C, P-O-P group vibrations in polysaccharide sugar rings of the cell wall polysaccharides and peptidoglycan (Fig 4D and 4H).

**Fig 5:**
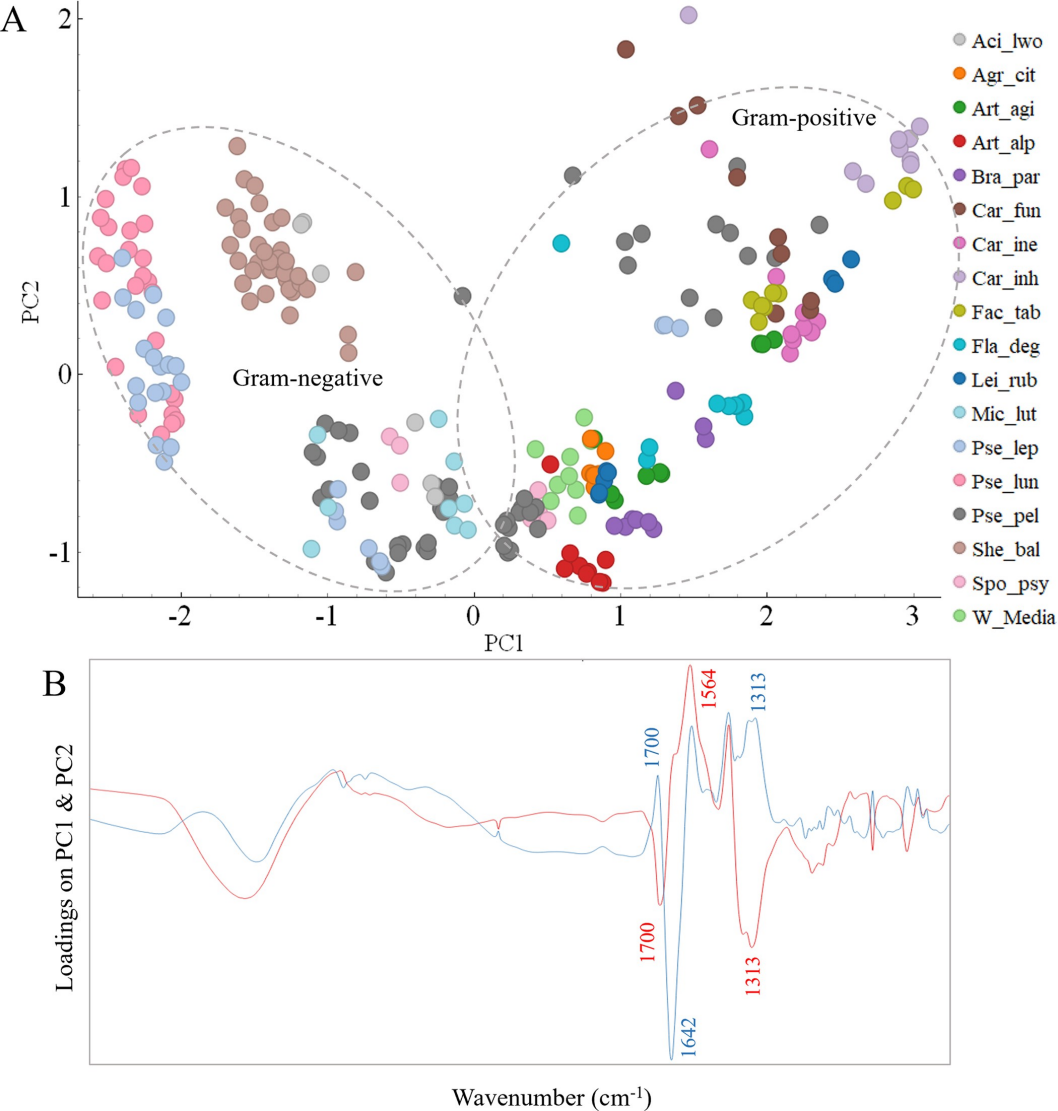
Principal component analysis (PCA) of the preprocessed FTIR spectra of supernatants obtained after cultivation of Antarctic bacteria in BHIB at 18°C. A–Score plot of PC1 and PC2 components, colors represent genera, shapes represent cultivation temperatures. B-Loading plot of FTIR data with main contributing peaks, PC1 (red) and PC2 (blue). PC1 provided 66% of explained variance and PC2 provided 15% of explained variance.

In addition to biomass, FTIR analysis of supernatants obtained after centrifugation of bacterial cultures grown in BHI broth was performed. The main characteristic peaks of FTIR spectra of pure BHI broth are C = O stretching in the proteins at 1645 cm^-1^ and 1570 cm^-1^, C = O symmetric stretching of COO- group in amino acids at 1400 cm^-1^ and peaks associated with phosphorus-containing compounds at 1083 cm^-1^ (S2 Fig). Analysis of supernatant spectra showed that bacteria from genus *Carnobacterium* and *Facklamia tabacinasalis* BIM B-1577 strain were characterized by an additional peak at 1570 cm^-1^ (S2 Fig). Additional peaks at 2338 cm^-1^, 835 cm^-1^ and 700 cm^-1^ were observed for *Shewanella baltica*, *Pseudomonas lundensis* and *Pseudomonas leptonychotis* species (S2 Fig). The PCA analysis of supernatant revealed a clear separation along PC1 for *Shewanella baltica* species from all other strains and another clear cluster was represented by *Pseudomonas lundensis* and *Pseudomonas leptonychotis* species (Fig 5A). The loading plot shows that the peaks of the protein region were responsible for this separation (Fig 5B).

### Impact of cultivation temperature on the cellular biochemical profile of meltwater bacteria

**Fig 6:**
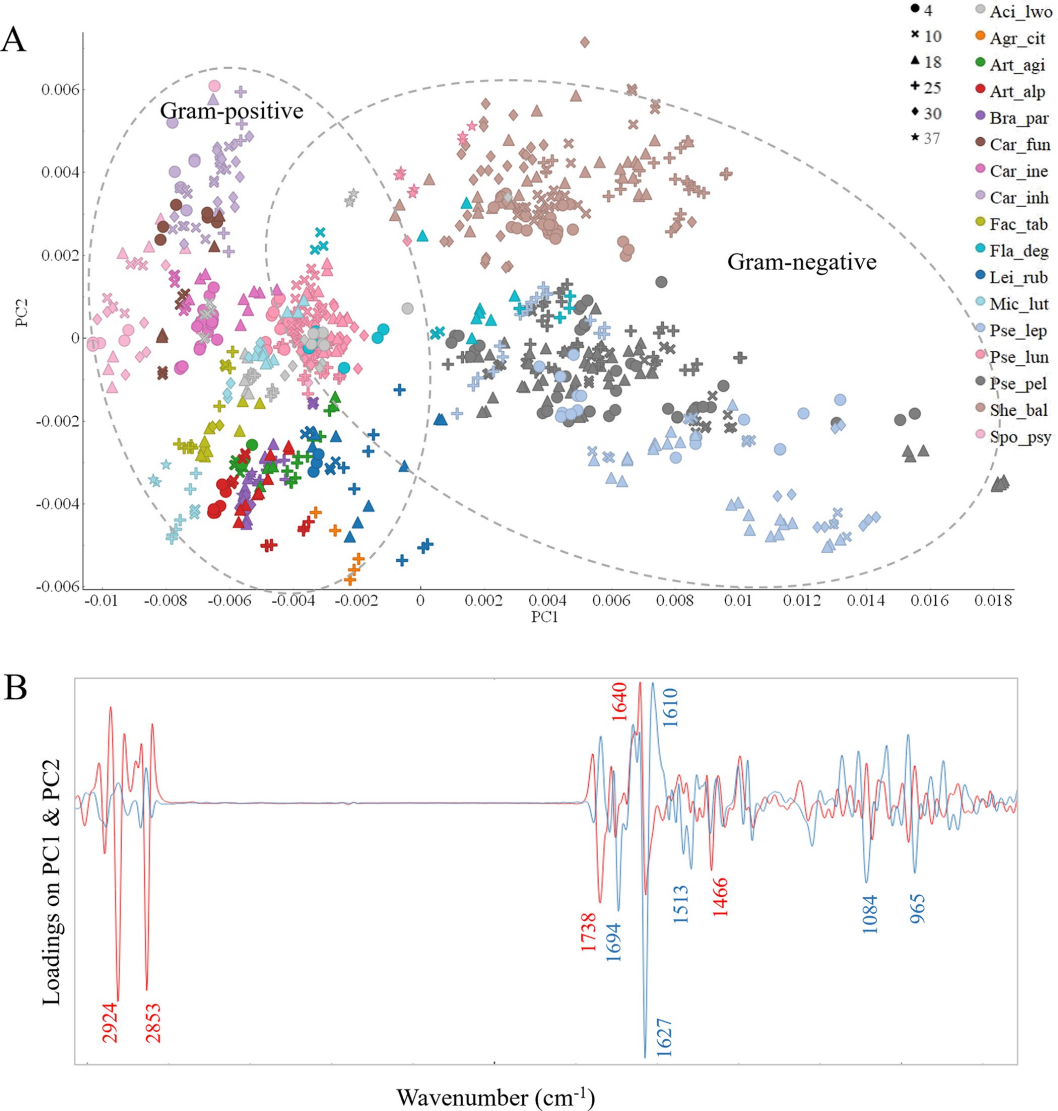
PCA of the preprocessed second derivative FTIR spectra of the Antarctic meltwater bacteria grown at different temperatures. **Shapes indicates different cultivation temperatures (‘●’– 4°C, ‘Ñ’– 10°C, ‘▲’– 18°C, ‘Ë’– 25°C, ‘◆’– 30°C, ‘★’– 37°C).** A–Score plot of PC1 and PC2 components, colors represent genera, shapes represent cultivation temperatures. B-Loading plot of the FTIR data with the main contributing peaks, PC1 (red) and PC2 (blue). PC1 provided 53% of explained variance and PC2 provided 12% of explained variance.

To investigate the impact of temperature on the total cellular biochemical profile of the Antarctic meltwater bacteria, we conducted cultivation experiments at various temperatures using BHIA medium. BHIA medium was chosen since it provided better clustering according to the taxonomy. The PCA analysis of the whole spectral region of the entire dataset of bacteria grown at different temperatures showed similar clustering as it was reported on Fig 6, where all Gram-negative bacteria except *Acinetobacter* and *Pseudomonas lundensis* strains, exhibited predominantly positive PC1 scores, suggesting higher lipid content in their cells, while Gram-positive bacteria predominantly displayed negative PC1 scores, indicating a higher protein content (Fig 6A and 6B). Samples that have negative scores have on average higher protein content represented by the positive peak at 1640 cm^-1^ in PC1 loading and samples that have positive scores have on average higher lipid content represented by the negative peaks at 2924 cm^-1^, 2853 cm^-1^, 1738 cm^-1^, 1466 cm^-1^. In addition, a clear separation along the PC2 axis was observed between strains *Pseudomonas leptonychotis* (BIM B-1559, BIM B-1568, BIM B-1566), *Pseudomonas peli* (BIM B-1560, BIM B-1569, BIM B-1546, BIM B-1552, BIM B-1542, BIM B-1548), *Flavobacterium degerlachei* BIM B-1562 and *Shewanella baltica* (BIM B-1565, BIM B-1557, BIM B-1561 and BIM B-1563), which according to the loading plot could be associated with the differences in proteins, phosphorus-containing molecules, and carbohydrates (Fig 6B).

Clear differences were observed in PC1 and PC2 between different species of bacteria cultivated at different temperatures (Fig 6A). Several bacterial species and strains formed isolated clusters when grown at different temperatures (Fig 6A):

- *Pseudomonas lundensis* BIM B-1554, BIM B-1555 and BIM B-1556 grown at 37°C (cluster 1) and samples grown at 4, 10, 18, 25, 30°C (cluster 2)
- *Flavobacterium degerlachei* BIM B-1562 grown at 4°C / 10°C (cluster 1) and 18°C / 25°C (cluster 2)
- *Micrococcus luteus* BIM B-1545 grown at 37°C and 25°C (cluster 1), 18°C and 30°C (cluster 2)
- *Acinetobacter lwoffii* BIM B-1558 grown at 18°C / 25°C (cluster 1), 4°C / 30°C (cluster 2) and 10°C (cluster 3)
- *Brachybacterium paraconglomeratum* BIM B-1571 grown at 10°C (cluster 1) and grown at (18, 25, 30 and 37°C)
- *Shewanella baltica* cultivated at all temperatures was the only species grouped separately from all other species with almost no overlapping.

A visual comparison of different spectral regions for the studied bacterial species indicated that lipid/ester region 1800–1700 cm^-1^ is relatively consistent and little affected by the temperature (S3 Fig). A change in the lipid region was observed for *Pseudomonas leptonychotis* strains and it was related to an increase in absorbance for the ester peak at 1742 cm^-1^ at low and extremely high temperatures. Further, slight increase of intensity for the peak at 1713 cm^-1^ associated with -C = O stretching in free fatty acids was observed for many bacteria grown at lower temperatures. For *Pseudomonas lundensis* strains and *Acinetobacter lwoffii* BIM B-1558 strains peak at 1713 cm^-1^ disappeared when bacteria were grown at 37°C (S3 Fig). This is an indication of increased production and possibly accumulation of free fatty acids with a temperature change. For the protein region 1700–1500 cm^-1^ the biggest effect of temperature in the form of shifts and change in intensity was detected for amide I peak at 1640 cm^-1^ and 1656 cm^-1^ related to β-sheet and α-helix structures of proteins, respectively. A shift to lower wavenumbers for the peak at 1640 cm^-1^ was detected for *Pseudomonas lundensis* strains *and Acinetobacter lwoffii* BIM B-1558 when grown at 37°C (S3 Fig), and an increase of protein-related peaks was detected for *Carnobacterium funditum* BIM B-1541 and *Carnobacterium iners* BIM B-1544 (S3 Fig).

The most significant temperature-triggered alterations were recorded in the mixed and polysaccharide spectral regions 1500–900 cm^-1^, where signals related to carbohydrates, nucleic acids and phosphates are present. Thus, an increase of intensity for the phosphodiester-related bands at 1240 cm^-1^ in mixed region and at 1083 cm^-1^ in polysaccharide region along with temperature decrease was recorded for majority of Gram-negative bacteria, while changes for Gram-positive bacteria were less intense with exception of *Arthrobacter agilis* BIM B-1543 (S3 Fig).

**Fig 7:**
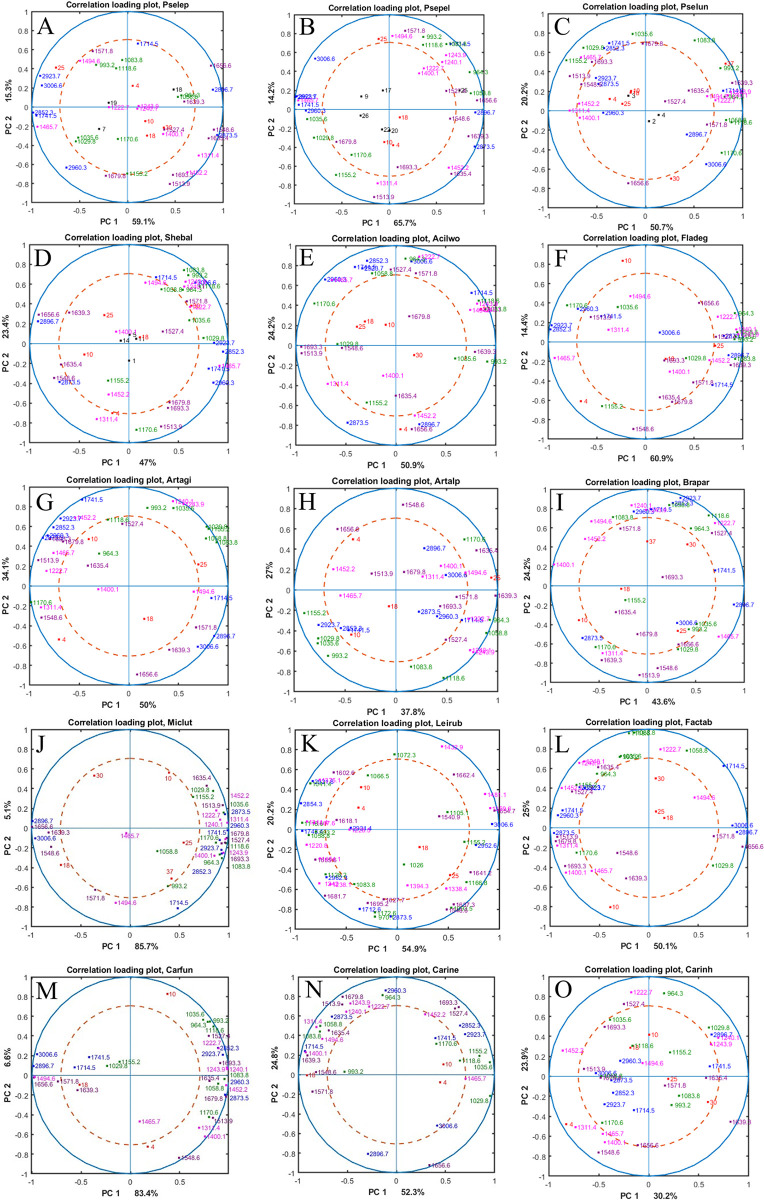
Correlation loading plots for PC1/PC2 for each species. Black—isolate number; red temperature; blue—lipid/ester region; pink—mixed region; violet—protein region, green—polysaccharide region.

The PCA correlation analysis was performed individually for each specie to reveal effect of temperature individually for each specie individually. The resulting correlation loading plots are presented on Fig 7. They illustrate correlations between spectral variables (major characteristic peaks) and design variables (temperature, strains when available).

Red and blue circles indicate the strength of the correlation in the corresponding PCs: correlation of 0.5 along the red circle and correlation of 1 along the blue circle, and no correlation for the variables close to the center of the correlation plot. The correlation between temperatures and spectral peaks was used to assess changes of cellular biochemical profile. In a correlation loading plot, the following types of correlation between variables can be found: (i) Positive correlation—when increase in one variable results in increases in another variable. Positive strong correlation is observed for the variables being close to each other and close to the blue circle. (ii) Negative correlation—when increase in one variable results in a decrease in another variable. Negative strong correlation is observed for the variables located opposite of each other and close to the blue circle. Variables located along PC1 and PC2 are not correlated, since PC1 is orthogonal to PC2.

For the majority of the studied bacterial species, at least one temperature (usually high or low temperature) showed to influence the biochemical composition. For example, for *Pseudomonas lundensis* strains (Fig 7C) 37°C contributed to the increase of carbohydrates (green peaks), especially the peak at 993 cm^-1^ and P-O-C symmetric stretching peak probably related to phospholipids at 1083 cm^-1^ and decrease of lipids (blue peaks) and proteins (purple). Interesting case of a high effect of temperature on the chemical profile was observed for *Flavobacterium degerlachei* BIM B-1562 (Fig 7F), where all temperatures seem to influence the biochemical composition quite strongly except 18°C. For this specie, 4 and 10°C negatively correlate to each other on PC2, while at 4°C we observed higher amounts of proteins (peaks at 1548, 1635, 1679 cm^-1^). 25°C is negatively correlated to 4°C and not correlated to 10°C on PC1 and PC2. At 25°C, there is an observable positive correlation with proteins, observed as elevated peaks at 1656 cm^-1^ and 1527 cm^-1^. Positive correlation for carbohydrates and mixed region detected for peaks at 964, 1058, and 993 cm^-1^ and peaks at 1240, 1243, and 1222 cm^-1^ respectively was predominantly associated with phosphodiester groups found in phospholipids (for Gram-negative bacteria) and teichoic/lipoteichoic acids (for Gram-positive bacteria). Notably, lipid production was decreasing at 25°C is as indicated by negative correlation with peaks at 2923 cm^-1^ and 2852 cm^-1^.

By analyzing correlation plots for all bacteria, it was noticed that the least effect on the biochemical profile for all studied species was at 18°C. This conclusion is drawn by assessing the proximity of each temperature point to the center of the correlation loading plot. The closer a point is to the center, the lesser its impact on PC1 and PC2. The growth at 25°C triggered changes in *Pseudomonas leptonychotis* strains (Fig 7A), *Pseudomonas peli* strains (Fig 7B), *Flavobacterium degerlachei* BIM B-1562 (Fig 7F), *Arthrobacter alpinus* BIM B-1549 (Fig 7H), *Arthrobacter agilis* BIM B-1543 (Fig 7G), where each species had specific responses but mainly associated with changes in lipids (blue peaks) and proteins (violet peaks). Interestingly, *Pseudomonas leptonychotis* and *Pseudomonas peli* had similar responses at 25°C, were we observed at this temperature higher amount of lipids and proteins was produced since it positively correlated with lipids peaks 3006 cm^-1^, 2923 cm^-1^, 1496 cm^-1^ and protein peak at 1571 cm^-1^. The growth at higher temperatures such as 30°C and 37°C affected only *Shewanella baltica* strains and *Pseudomonas lundensis*, *Acinetobacter lwoffii* BIM B-1558 strains (Fig 7D, 7C and 7E, respectively), where the changes were associated mainly with proteins (violet peaks) and polysaccharides (green peaks) (Fig 7D, 7C and 7E respectively). Low temperatures (4°C and 10°C) were shown to have the highest effect on the biochemical profile for majority of the studied species, except all *Pseudomonas* species, *Arthrobacter alpinus* BIM B-1549, *Leifsonia* sp. BIM B-1567 and *Carnobacterium iners* BIM B-1544, where each species showed specific responses. Low growth temperatures were associated with higher peaks at 1311, 1452 cm^-1^ from mixed region (pink), 1548 cm^-1^ from protein region (violet) and 1170 cm^-1^, 1155 cm^-1^ from polysaccharide region (green) (Fig 7). All tested temperatures had considerable effect on the biochemical profiles of *Micrococcus luteus* BIM B-1545 (Fig 7J) and all of *Carnobacterium* species. Correlation analysis showed that the highest and the lowest temperatures forced the bacteria to adapt to the condition causing the most significant changes in biochemical profile.

## Discussion

Characterization of the Antarctic meltwater bacteria by FTIR spectroscopy showed biochemical differences of these bacteria on various taxonomic levels and the most obvious differences were observed for different Gram groups, which showed considerable variation in lipid region. These results are in accordance with the previously reported and can be explained by the fact that Gram-positive bacteria have naturally higher peptidoglycan content, whereas Gram-negative bacteria have higher lipid content [64,70,71]. Gram-negative bacteria have an outer membrane, in addition to their inner membrane, which is composed of lipopolysaccharides and phospholipids that can contribute to the higher total lipid content. Second noticeable difference between two Gram groups was related to the peaks associated with the phosphodiester group present in various molecules, such as DNA, phospholipids and teichoic acids and lipoteichoic acids [64]. In Gram-positive bacteria, these peaks seem to be associated with mainly teichoic acids and lipoteichoic acid due to a low amount of phospholipids [64]. In addition, FTIR analysis revealed differences in protein structure between two Gram groups. The studied bacteria are psychrotrophic but, according to the literature, the same differences between Gram-negative and Gram-positive bacteria characterized as mesophilic can be expected [8,64].

In addition to Gram classification, FTIR profiling provided clear clustering on genus and species level that was also well aligned with taxonomy. Cultivation on agar provided better taxonomy-aligned clustering than cultivation in broth media. Also, chemical variability was much lower for bacterial biomass obtained from agar cultivation than from broth cultivation. This can be due to the fact that agar-based cultivation is static and characterized by the consistency of conditions such as oxygen availability and temperature, while cultivation in broth can vary in oxygen accessibility and overall gas transfer [72]. Further, the total cellular biochemical profile of bacteria grown on agar and broth differed considerably especially for some spectral regions such as polysaccharide region. Interestingly, lipid spectral region was little affected by different forms of cultivation medium and allowed clear taxonomy-aligned clustering on genus and species levels. This might be an indication that lipids are the least affected by the cultivation media type. This justifies the fact that fatty acid profile of lipids is used as classification biomarker for chemotaxonomy of bacteria [73].

In polar regions, temperature is a factor considerably affecting microbiota [74] and it plays a crucial role in developing adaptation mechanisms in microbes inhabiting these regions [75]. Therefore, in this study we investigated the impact of temperature on the total cellular biochemical profile of the Antarctic meltwater bacteria. The observed results indicate that temperature impact is species-specific and variation of biochemical profile for different strains within a single species is smaller than variations caused by temperature as was also shown previously [48]. For the majority of the studied bacteria, the highest impact on cell chemistry was on upper and lower limits of growth temperatures. We observed that for many studied strains, proteins and especially polysaccharides were considerably affected by temperature. This could indicate that proteins and polysaccharides play a key role in temperature adaptation and survival of bacteria in extreme environments [76]. The change in the protein levels of Antarctic bacteria is more likely associated with the metabolic activity of the cells and the functionality of enzymes in cold-adapted bacteria such us increased activity and flexibility [77]. Lipids were less affected by the temperatures in comparison to proteins and polysaccharides. However, for some bacteria, such as for example *Pseudomonas leptonychotis*, an increase of absorbance for ester peak at 1743 cm^-1^ was observed when grown at low and high temperatures and may indicate about production of PHA [78]. Many bacteria accumulate PHAs as carbon and energy reserves, often in response to limited essential nutrients in the growth medium, although some produce PHAs during growth without such conditions. PHA serves as an alternate source of fatty acids, vital for their survival under stress conditions [79,80].

Correlation loading plots facilitate the visualization of loading plots, particularly when utilizing design variables. Overall, correlation loading plots showed that the biggest temperature impact was for the bacteria with the wide growth temperature range and able to grow at 4 to 37°C such as *Pseudomonas lundensis* strains and *Acinetobacter lwoffii* BIM B-1558 since low and high growth temperatures for this strains cluster close to the circle edge of a correlation loading plot, it suggests a higher degree of correlation with the principal components being studied.

## Conclusion

This study, for the first time, reports the effect of the growth conditions on the cellular biochemical profile of the Antarctic meltwater bacteria. The findings indicate that utilizing agar-based BHI medium is the preferred choice for comprehending the biochemical nature of phylogenetic relations and examining the influence of abiotic factors on bacterial cell chemistry. This preference is due to the reduced variability between spectra observed during cultivation on agar, as compared to broth-based cultivation. Species-specific temperature-induced changes in the total cellular biochemical profile were observed, with the most significant effects seen in bacteria exhibiting a broad growth temperature range. Correlation analysis further revealed that these bacteria tend to undergo the most substantial changes in their biochemical profile when exposed to either the highest or lowest temperatures they can resist. Furthermore, the study found that alterations in lipids and proteins due to temperature were less pronounced and detected only in few species while changes in polysaccharides were more common for all bacteria. Overall, FTIR spectroscopy for bacterial profiling offers a promising approach for efficiently screening the impact of cultivation conditions in high-throughput settings. This study, to the authors knowledge, is the first to reveal the comparison of form of cultivation media and temperature effect on bacteria cells. It highlights that polysaccharides are the most flexible chemical components of the cell involved in adaptation.

## Supporting information

S1 Fig Preprocessed second derivative FTIR-HTS spectra averaged for agar and broth media of each species. (TIF)

S2 Fig Preprocessed FTIR-HTS spectra of BHIB media and spectra of supernatant for each species. (TIF)

S3 Fig Second derivative FTIR spectra of bacterial biomass of different bacterial species grown at different temperatures. Colors indicate cultivation temperatures (blue– 5°C, dark blue– 10°C, green– 18°C, orange– 25°C, pink– 25°C and red– 25°C).
(TIF)

S1 Table Overview over cultivation time (days), temperatures and growth of the Antarctic meltwater bacteria cultivated on BHIB and BHIA. Gray color indicates the absence or very little growth.
(PDF)

S1 File (PDF)

S1 Graphical abstract (TIF)
