# Supplementary figures and images for: Global biochemical profiling of fast-growing Antarctic bacteria isolated from meltwater ponds by high-throughput FTIR spectroscopy
Source: PLoS One. 2024 Jun 17;19(6):e0303298. doi: 10.1371/journal.pone.0303298 (PMC11182503; doi:10.1371/journal.pone.0303298)

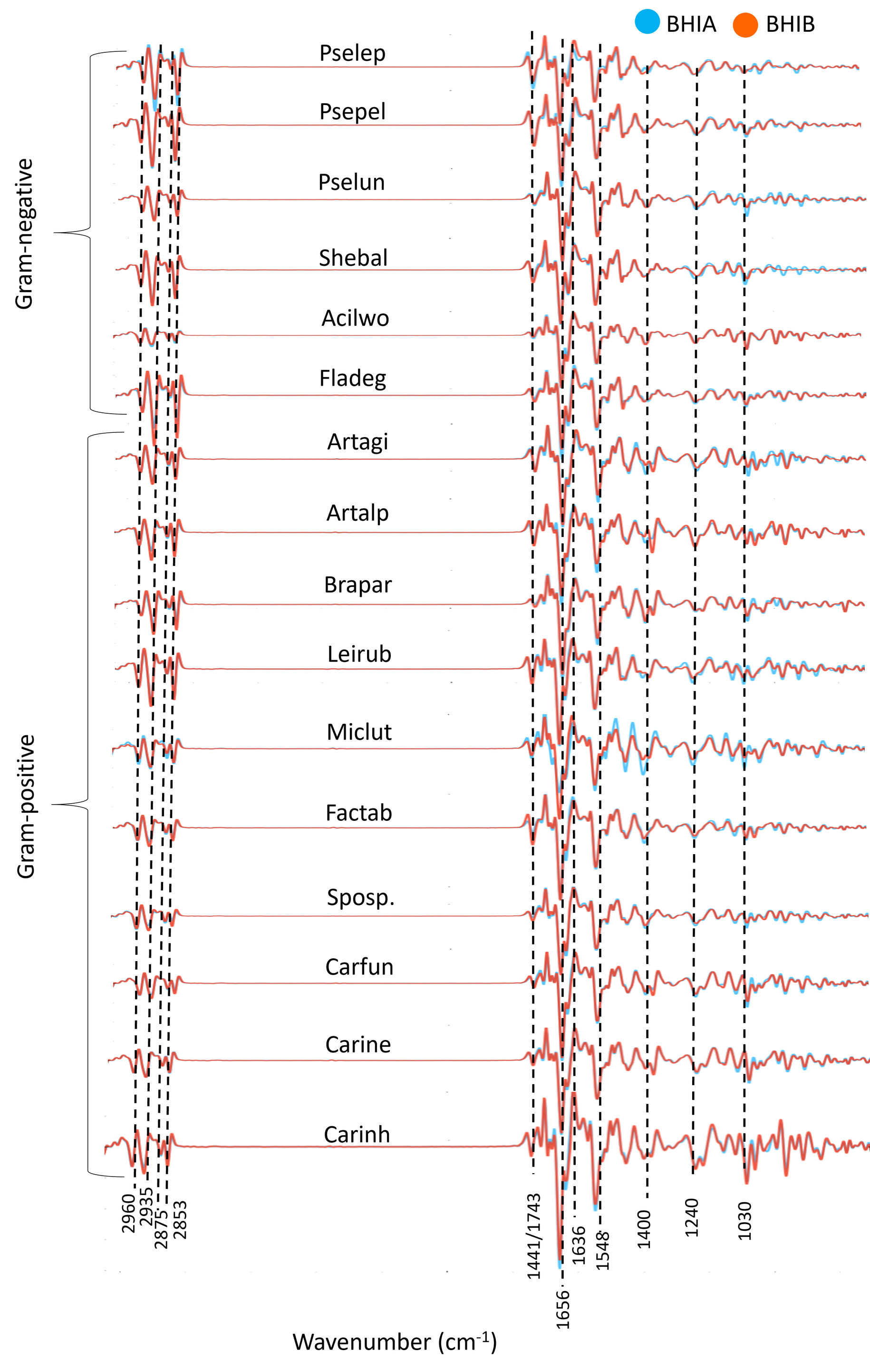

Supplement: S1 Fig — (TIF) [file pone.0303298.s001.tif]

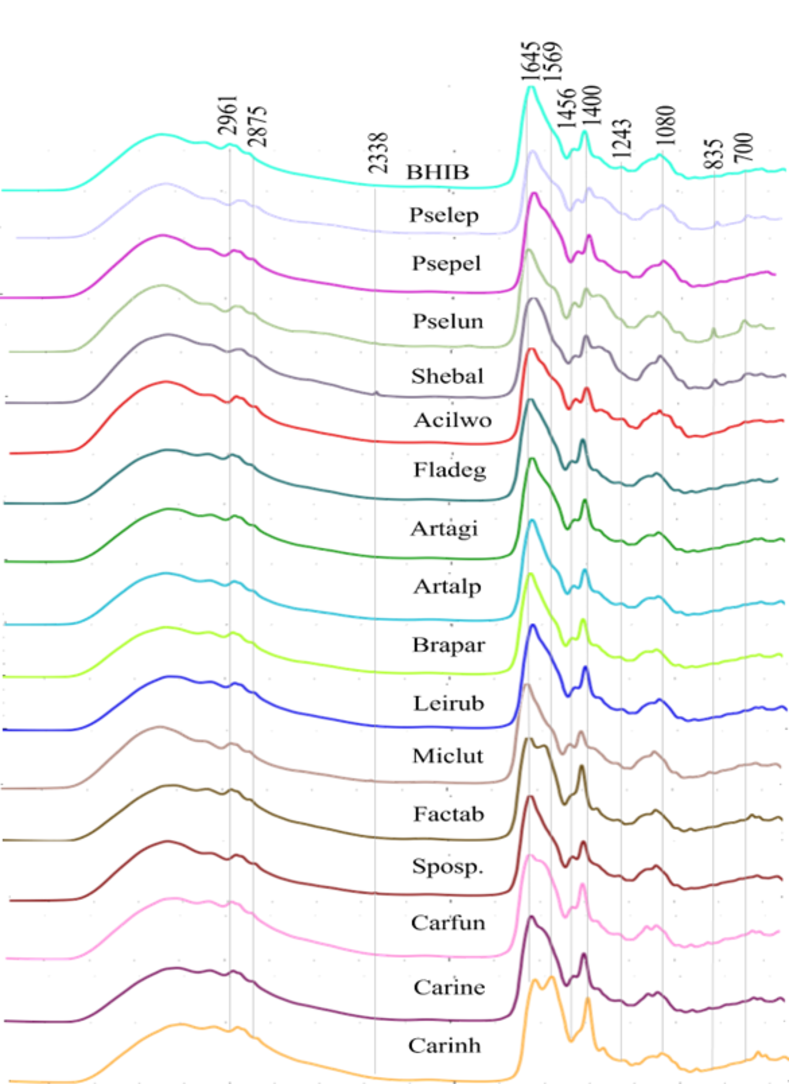

Supplement: S2 Fig — (TIF) [file pone.0303298.s002.tif]

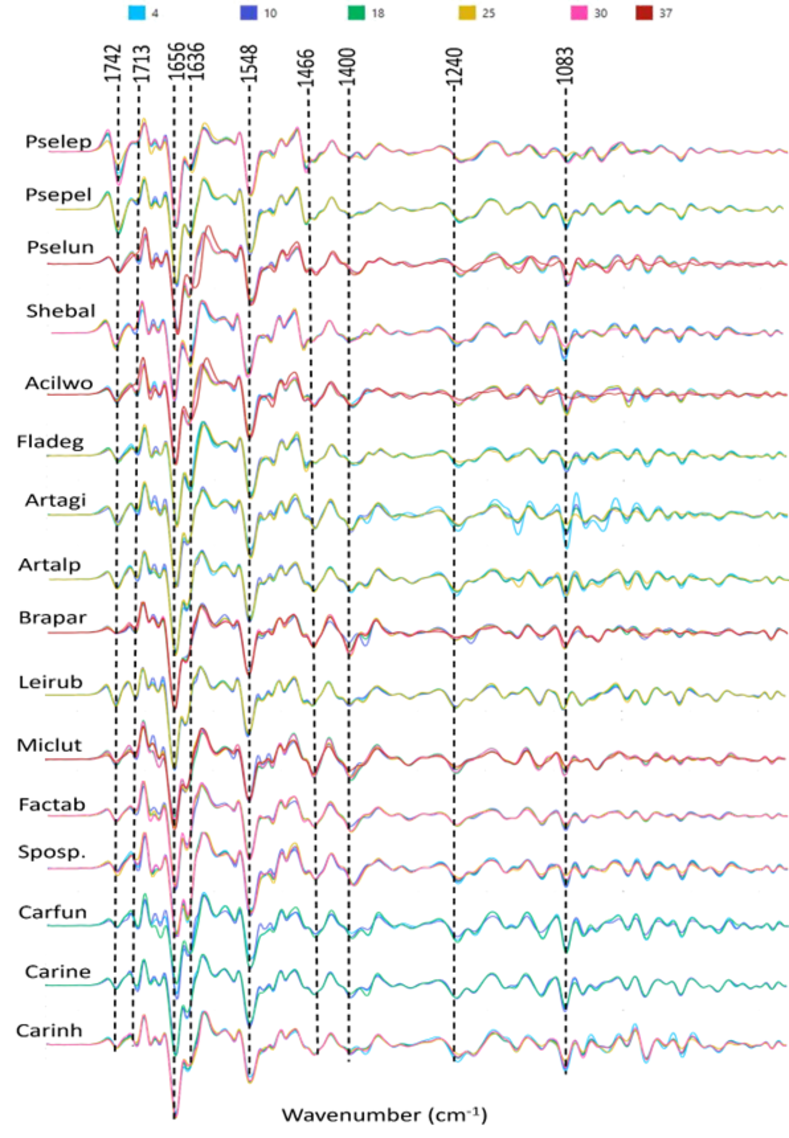

Supplement: S3 Fig — Colors indicate cultivation temperatures (blue– 5°C, dark blue– 10°C, green– 18°C, orange– 25°C, pink– 25°C and red– 25°C). (TIF) [file pone.0303298.s003.tif]

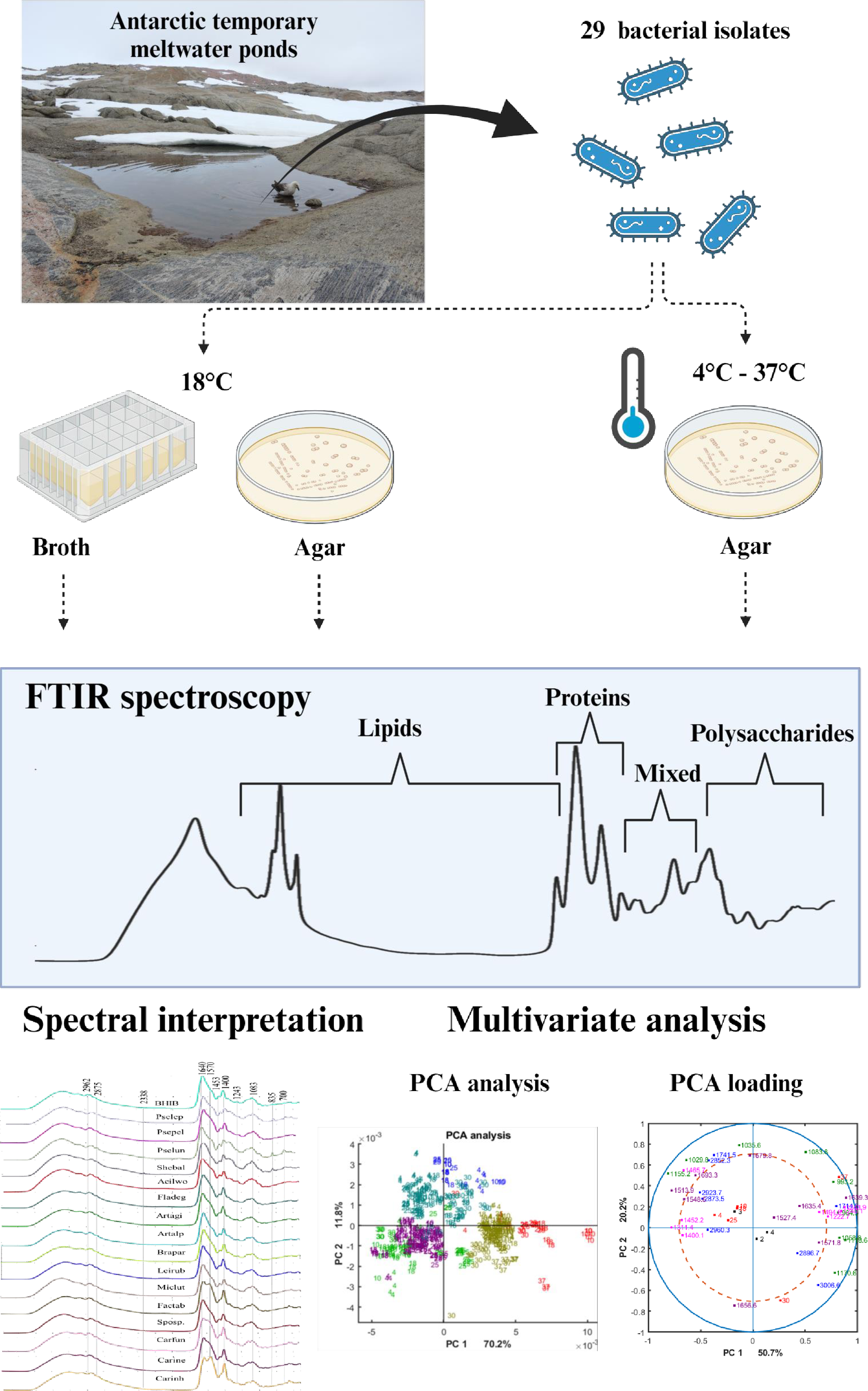

Supplement: S1 Graphical abstract — (TIF) [file pone.0303298.s006.tif]
