# Supplementary material for: Global biochemical profiling of fast-growing Antarctic bacteria isolated from meltwater ponds by high-throughput FTIR spectroscopy
Source: PLoS One. 2024 Jun 17;19(6):e0303298. doi: 10.1371/journal.pone.0303298 (PMC11182503; doi:10.1371/journal.pone.0303298)
Supplement: S1 Table — Gray color indicates the absence or very little growth. (PDF) [file pone.0303298.s004.pdf]

S1 Table. Overview over cultivation time (days), temperatures and growth of the Antarctic meltwater bacteria cultivated on BHIB and BHIA. Gray color indicates the absence or very little growth

Genus	Specie	Short abbreviation	Strain	Collection number	BHIB	BHIA					
					18°C	4	10	18	25	30	37
**Gram-negative**											
Shewanella	*Shewanella baltica*	Shebal	TMP1	BIM B – 1565	3	4	4	2	1	1	
			TMP5	BIM B – 1557	3	4	4	2	1	2	
			TMP11	BIM B – 1561	3	4	4	2	1	2	
			TMP14	BIM B – 1563	3	4	4	2	1	1	
Acinetobacter	*Acinetobacter lwoffii*	Acilwo	TMP6	BIM B – 1558	3	16	4	2	1	2	3
Pseudamonas	*Pseudomonas lundensis*	Pselun	TMP2	BIM B – 1554	3	4	4	2	1	1	3
			TMP3	BIM B – 1555	3	4	4	2	1	1	3
			TMP4	BIM B – 1556	3	4	4	2	1	1	3
	Pseudomonas leptonychotis	Pselep	TMP7	BIM B – 1559	3	6	4	3	3	4	
			TMP18	BIM B – 1568	3	6	3	3	3	2	
			TMP19	BIM B – 1566	3	6	3	3	3	4	
	Pseudomonas peli	Psepel	TMP9	BIM B – 1560	3	9	4	3	3		
			TMP17	BIM B – 1569	3	6	5	3	3		
			TMP20	BIM B – 1546	3	9	4	3	3		
			TMP22	BIM B – 1552	3	6	4	4	3		
			TMP25	BIM B – 1542	3	6	5	5	3		
			TMP26	BIM B – 1548	3	6	4	3	3		
Flavobacterium	*Flavobacterium degerlachei*	Fladeg	TMP13	BIM B – 1562	3	6	5	5	3		
**Gram positive**											
Sporosarcina	*Sporosarcina* sp.	Sposp	TMP10	BIM B – 1539	3	6	4	3	3	2	
Carnobacterium	*Carnobacterium funditum*	Carfun*	TMP27	BIM B – 1541	3	6	5	5			
	Carnobacterium iners	Carine*	TMP28	BIM B – 1544	3	9	4	5			
	Carnobacterium inhibens	Carinh	TMP12	BIM B – 1540	3	6	7	4	2	2	
Facklamia	*Facklamia tabacinasalis*	Factab	TMP29	BIM B – 1577	3		5	5	4	4	
Arthrobacter	*Arthrobacter* sp.	Artcit	TMP15	BIM B – 1549	3	4	4	3	3		
	Arthrobacter agilis	Artagi	TMP24	BIM B – 1543	3	9	6	3	4		
Brachybacterium	*Brachybacterium paraconglomeratum*	Brapar	TMP16	BIM B – 1571	3		5	4	3	2	3
Micrococcus	*Micrococcus luteus*	Miclut	TMP21	BIM B – 1545	3		6	3	3	1	3
Agrococcus	*Agrococcus citreus*	Agocit	TMP23	BIM B – 1547	3				4		
Leifsonia	*Leifsonia* sp.	Leisp	TMP30	BIM B – 1567	3	8	5	4	4		
