# Supplementary material for: Global biochemical profiling of fast-growing Antarctic bacteria isolated from meltwater ponds by high-throughput FTIR spectroscopy
Source: PLoS One. 2024 Jun 17;19(6):e0303298. doi: 10.1371/journal.pone.0303298 (PMC11182503; doi:10.1371/journal.pone.0303298)
Supplement: S1 File — (PDF) [file pone.0303298.s005.pdf]

Genus	Species	Short abbreviation	Strain	Collection number	BHIB	BHIA					
					18°C	4	10	18	25	30	37
**Gram-negative**											
Shewanella	*Shewanella baltica*	Shebal	TMP1	BIM B – 1565	3	4	4	2	1	1	
			TMP5	BIM B – 1557	3	4	4	2	1	2	
			TMP11	BIM B – 1561	3	4	4	2	1	2	
			TMP14	BIM B – 1563	3	4	4	2	1	1	
Acinetobacter	*Acinetobacter lwoffii*	Acilwo	TMP6	BIM B – 1558	3	16	4	2	1	2	3
Pseudomonas	*Pseudomonas lundensis*	Pselun	TMP2	BIM B – 1554	3	4	4	2	1	1	3
			TMP3	BIM B – 1555	3	4	4	2	1	1	3
			TMP4	BIM B – 1556	3	4	4	2	1	1	3
	Pseudomonas leptonychotis	Pselep	TMP7	BIM B – 1559	3	6	4	3	3	4	
			TMP18	BIM B – 1568	3	6	3	3	3	2	
			TMP19	BIM B – 1566	3	6	3	3	3	4	
	Pseudomonas peli	Psepel	TMP9	BIM B – 1560	3	9	4	3	3		
			TMP17	BIM B – 1569	3	6	5	3	3		
			TMP20	BIM B – 1546	3	9	4	3	3		
			TMP22	BIM B – 1552	3	6	4	4	3		
			TMP25	BIM B – 1542	3	6	5	5	3		
			TMP26	BIM B – 1548	3	6	4	3	3		
Flavobacterium	*Flavobacterium degerlachei*	Fladeg	TMP13	BIM B – 1562	3	6	5	5	3		
**Gram-positive**											
Sporosarcina	*Sporosarcina* sp.	Sposp	TMP10	BIM B – 1539	3	6	4	3	3	2	
Carnobacterium	*Carnobacterium funditum*	Carfun*	TMP27	BIM B – 1541	3	6	5	5			
	Carnobacterium iners	Carine*	TMP28	BIM B – 1544	3	9	4	5			
	Carnobacterium inhibens	Carinh	TMP12	BIM B – 1540	3	6	7	4	2	2	
Facklamia	*Facklamia tabacinasalis*	Factab	TMP29	BIM B – 1577	3		5	5	4	4	
Arthrobacter	*Arthrobacter alpinus*	Artalp	TMP15	BIM B – 1549	3	4	4	3	3		
	Arthrobacter agilis	Artagi	TMP24	BIM B – 1543	3	9	6	3	4		
Brachybacterium	*Brachybacterium paraconglomeratum*	Brapar	TMP16	BIM B – 1571	3		5	4	3	2	3
Micrococcus	*Micrococcus luteus*	Miclut	TMP21	BIM B – 1545	3		6	3	3	1	3
Agrococcus	*Agrococcus citreus*	Agrcit	TMP23	BIM B – 1547	3				4		
Leifsonia	*Leifsonia* sp.	Leisp	TMP30	BIM B – 1567	3	8	5	4	4		
